# Intranigral Injection of Endotoxin Suppresses Proliferation of Hippocampal Progenitor Cells

**DOI:** 10.3389/fnins.2019.00687

**Published:** 2019-07-03

**Authors:** Batoul Darwish, Farah Chamaa, Elie D. Al-Chaer, Nayef E. Saadé, Wassim Abou-Kheir

**Affiliations:** Department of Anatomy, Cell Biology and Physiological Sciences, Faculty of Medicine, American University of Beirut, Beirut, Lebanon

**Keywords:** neurogenesis, neuro-inflammation, dentate gyrus, substantia nigra, progenitors, endotoxin

## Abstract

Brain inflammation can result in functional disorders observed in several neurodegenerative diseases and that can be also associated with reduced neurogenesis. In this study, we investigate the effect of mild inflammation, induced by unilateral injection of Endotoxin (ET) in the substantia nigra (SN)/Ventral Tegmental Area, on the proliferation and survival of stem/progenitor cells in the dentate gyrus (DG) of the hippocampus. Adult female rats received unilateral injection of ET (2 μg/2 μl saline) or sterile saline (2 μl) in the right SN followed by 5′-Bromo-2′-deoxyuridine (BrdU) injections (66 mg/kg/injection). Intranigral ET injection induced bilateral decrease in the number of newly born BrdU positive cells in the DG. This effect was paralleled by a significant decrease in the exploratory behavior of rats, as assessed by the Y-maze novel arm exploration task. ET also induced a transient decrease in the number of tyrosine hydroxylase-positive cells in the injected SN, impaired motor behavior, and caused microglial activation in the SN. This study provides an experimental simulation of the remote effects of moderate and reversible neuroinflammation resulting in impaired communication between midbrain dopaminergic neurons and the hippocampus.

## Introduction

The substantia nigra (SN) is the main region affected in Parkinson disease (PD) which is characterized by the progressive loss of dopaminergic neurons in that region leading to motor deficits and ultimately to impaired cognitive behavior (Hritcu and Ciobica, 2013). Non-motor symptoms of PD usually appear years before the onset of motor symptoms and these include anhedonia, depression, anxiety and impaired performance upon novelty exposure. It is worth noting that more than half of the SN dopaminergic neurons are lost before the onset of motor symptoms indicating a possible relation between early dopaminergic loss and changes in cognitive function and behavior (Winkler et al., 2011). Dopaminergic neurons from the SN and ventral tegmental area (VTA) project to the hippocampus in a topographically organized manner (Gasbarri et al., 1994, 1997). These connections are both ipsilateral and contralateral to the hippocampus, with the contralateral connections coming mainly from the VTA. Such dopaminergic input is thought to be important for directing the choice of memory encoding in the hippocampus and therefore might have influence on its neurogenic niche (Du et al., 2016; Duszkiewicz et al., 2018). Dopaminergic neurons from the VTA and SN pars compacta (pc) innervate the lower and upper blade of the DG supplying newly born DG cells (Hoglinger et al., 2014). Moreover, dopamine is important for promoting the survival of new born neurons in the DG (Takamura et al., 2014). There is an established role for dopamine and dopaminergic input from the SN and VTA in the regulation of adult neurogenesis. Other structures of the hippocampus such as the subiculum and the CA1 region mainly receive afferents from the medial part of the SNpc (Gasbarri et al., 1994).

Advanced imaging (Carlesimo et al., 2012) and postmortem studies of PD (Hoglinger et al., 2004) show that adult neurogenesis is compromised in PD patients although the exact mechanisms leading to this are not fully understood. It has been postulated that altered hippocampal neurogenesis plays a crucial role in PD depression (Lim et al., 2018). Furthermore, impaired hippocampal neurogenesis has been reported in animal models of PD (Marxreiter et al., 2013; Sung, 2015; Klein et al., 2016) and has been correlated with Non-motor symptoms of PD pathogenesis that include spatial learning and memory (Pillon et al., 1989; Lamm et al., 2014; Regensburger et al., 2014). It is worth noting, however, that most of these studies were essentially based on experimental models that used toxic substances like MPTP (1-methyl-4-phenyl-1,2,3,6-tetrahydropyridine) and 6-hydroxydopamine (6-OHDA) producing irreversible loss of dopaminergic neurons (Gasbarri et al., 1994, 1997; Hoglinger et al., 2004, 2014). Therefore, using inflammogens might offer a better simulation of the pathological conditions in neurodegenerative processes.

Inflammation in the central nervous system (CNS) is commonly associated with a number of neurodegenerative disorders, such as Parkinson, Alzheimer’s and Huntington diseases (Tufekci et al., 2012). Furthermore, inflammation has already been established as one of the factors that can impair neurogenesis (Ekdahl et al., 2003; Chamaa et al., 2018b). Lipopolysaccharide (LPS) (or endotoxin) is a potent inflammatory substance that exerts its effects through activating microglial cells and through the release of pro-inflammatory mediators (Lieberman et al., 1989; Safieh-Garabedian et al., 2011; Chamaa et al., 2018b). It has been previously established that intranigral LPS injection induces damage in the SN dopaminergic system (Castano et al., 1998, 2002; Herrera et al., 2000; Hoban et al., 2013; Reinert et al., 2014). However, the impact of such inflammation on hippocampal neurogenesis has not been fully examined. Therefore, the aim of this study was to investigate the effect of mild acute inflammatory insult in the SN and its possible extended effect on the hippocampus, in particular, the proliferation and survival of neural stem/progenitor cells (NSPCs) in the dentate gyrus and its possible impact on rat’s exploratory behavior.

## Materials and Methods

### Animals

A total of 35 female Sprague-Dawley rats (250–300 *g*) were used in this study. The rats were housed under standard colony conditions in a room maintained at a constant temperature (20–22°C) on a 12 h light/dark cycle with standard rodent chow and water provided *ad libitum*. Surgical procedures were conducted under deep anesthesia by intraperitoneal (*ip*) injection of ketamine (Ketalar^®^; 50 mg/kg) and xyla (Xylazine^®^; 12 mg/kg). Postoperative surveillance of the behavior and body weight of the rats was performed daily during the light phase of the cycle. All experimental procedures were approved by the Institutional Animal Care and Use Committee.

### Intra-Nigral Injection of ET

**FIGURE 1:**
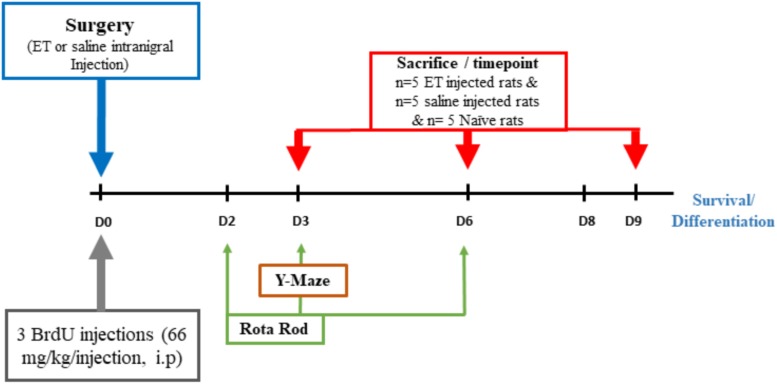
ExperimentalParadigm. Flow chart summarizing the timing of the different experimental procedures.

The head of the anesthetized rat was firmly fixed in a stereotaxic frame (Kopf, CA). The scalp was shaved, and a small skin incision was made to expose the skull. A hole was drilled into the skull to allow the penetration of a 10 μl Hamilton Syringe needle (Hamilton Company, Nevada, United States) above the targeted stereotaxic coordinates of the right SN (lateral: 1.5 mm; vertical: 8 mm and anterior–posterior: –5.5 mm in reference to bregma (Paxinos and Watson, 1998). Each injection was made of either 2 μl of endotoxin (ET) (Lipopolysaccharide from Salmonella typhosa; Sigma) or 2 μl of sterile saline for the sham group. ET was dissolved at a concentration of 1 μg/μl saline and 2 μl were injected in two boluses of 1 μl each, given 1 min apart. Rats were distributed in 2 sets: sham injected with saline (*n* = 15), or ET injected (*n* = 15). Based on previous studies, brain tissue was collected from rats in the three groups at three time points after the ET injection: day 3 (*n* = 5/group), day 6 (*n* = 5/group) and day 9 (*n* = 5/group) (Castano et al., 1998, 2002; Arimoto et al., 2007). Additionally, a total of 5 naïve not operated on rats (*n* = 5) were used. A summary of the chronology of different experimental procedures is given in Figure 1.

### BrdU Administration

The synthetic thymidine analog 5′-bromo-2′-deoxyuridine Bromodeoxyuridine (BrdU) powder (Sigma-Aldrich) was weighed and dissolved in 0.9% warm saline. Three BrdU injections were administered to each rat on the same day of the procedure (including naïve and sham rats) with 2 h intervals between consecutive injections (66 mg/kg per 300 μl injection, *ip*) (Figure 1).

### Behavioral Tests

#### Rota Rod Test

The Rota rod test was performed to assess motor performance and balance using a commercially available rat apparatus (fixed speed at 5 rpm). In the training session, that was done 1 day ahead of experiments, each rat was placed on the rotating rod for familiarization. Rats were given three independent trials daily. Each trial lasted for 120 s and was separated by a 10 min inter-trial period. The latency to fall off the rotating rod was recorded. The test was performed on days 1, 2, 3 and 6 after ET injection. The results were expressed as the average retention time of the three test trials during each session.

#### Y-Maze Test

Y-maze was used for novel arm discrimination to assess hippocampal-dependent spatial memory. This test mainly examines the innate behavior of rats to explore a novel area more than a familiar one. The Y-maze apparatus consists of three identical arms (10 cm wide and 40 cm long). No intra-maze cues were added. The test mainly consisted of two phases; a 15 min training or acquisition phase and an experimental 5 min phase separated by a 1 h- interval. In the first training trial, one of the arms, labeled as the novel arm, was blocked and rats were placed in the “Start” arm and allowed to explore it along with the third remaining arm. In the experimental test, the closed/novel arm (N) was opened and rats were placed in the Start arm and allowed to roam and explore the three arms for 5 min whereby the total time spent in each arm and the number of entries to each arm were recorded. The floor and walls of the maze were wiped with 70% alcohol at the end of trial with each rat to avoid odor cues. This test was conducted on day 3 following intranigal injection of either ET or sterile saline.

### Brain Tissue Sampling and Processing

Anesthetized rats were intracardially perfused with saline followed by 4% paraformaldehyde in saline. The brains were removed, post-fixed in the same fixative overnight and immersed in 30% sucrose for 48 h at 4°C for cryoprotection. They were then coronally sectioned at 40 μm intervals with a freezing microtome and were stored in 15 mM sodium azide in PBS at 4°C in 24 well plates. Systemic sampling of the brain sections was achieved following the fractionator method for unbiased stereology (Schmitz et al., 2014; Chamaa et al., 2018a). In brief, the first section collected was placed in the first well, the second section in the second well until the sixth section is reached. The seventh section was then put back in the first well and so on. At the end, each one of the six wells would be a representative of the region that was cut and one well would be chosen randomly out of the six for immunofluorescence. The optical fractionator technique is designed to provide estimates of the total number of cells from thick sections taken from a particular region (Olesen et al., 2017). The DG was divided into three areas as follows: rostral ranging from − 2.12 to − 3.7 mm relative to bregma, intermediate ranging from − 3.7 to − 4.9 mm and caudal ranging from−4.9 to −6.3 mm (Chamaa et al., 2016, 2018a). Sections were serially collected in 6 sets containing 7 rostral, 5 intermediate and 6 caudal sections per set. The SN region was collected with the caudal DG sections as it lies within the same coordinates (−4.8 to −6.3 mm).

### Immunofluorescence and Confocal Microscopy

One representative well of each topographic region was chosen randomly for the assessment of BrdU uptake by progenitor cells. Tissues were then incubated at 37°C with 2N HCL for 30 min to denature the DNA and allow the optimal binding of the antibody to the previously incorporated BrdU. Sections were washed once with PBS and then with Sodium borate (0.1 M, 8.5 PH) for 10 min at room temperature in order to neutralize the acidity. This was followed by three washes with PBS before the sections could be transferred to a freshly prepared blocking solution (10% NGS, 10% BSA and 0.1% Triton X diluted in PBS) for 1 h at 4°C. After that, they were incubated overnight at 4°C with the primary antibody anti-BrdU (1/100; Bio-Rad) diluted in 3% NGS, 3% BSA and 0.1% Triton X. The next day, tissues were washed and incubated in the dark with secondary antibody goat anti-rat Alexa-Fluor 568 (1/100; Invitrogen) for 2 h at RT on a shaker. They were then washed and incubated with the second primary antibody NeuN (1/500; Millipore) overnight and on the following day with secondary antibody Alexa-Fluor 488 goat anti-mouse (1/250; Invitrogen) as previously mentioned. Three washes were followed and Hoechst stain (1/10000; Invitrogen) was added. The sections were then mounted with a brush on labeled slides, mounting media was added and slides were covered with a thin glass coverslip. Moreover, we further stained for ki67 (1/500; Abcam), DCX (1/500; Abcam) and GFAP (1/500; Abcam) with their respective secondary antibody Alexa-Fluor 488 goat anti- rabbit (1/250; Invitrogen).

For microglia and Tyrosine Hydroxylase (TH) staining, tissues were washed with PBS, blocked with the same blocking solution as previously mentioned and incubated with: anti-CD11b/c Clone OX-42 (1/50; BD Pharmingen) or anti-TH (1/500, Abcam) overnight at 4°C. The next day, tissues were washed and incubated with secondary antibodies.

Confocal microscopy (Zeiss LSM 710) was used for acquiring images and data quantification. Tile scan and serial *z*-stacks for BrdU with maximal intensity projection were taken at 40× oil objective. CD11b/c signal intensity was quantified using Zeiss ZEN 2009 image-analysis software where the same settings were used for the different conditions and measurements were done in a constant area for all sections.

### Cell Quantification

Detection of stem/progenitor BrdU positive cells in the SGZ was determined using confocal microscopy on the 40×-oil objective. The counting was done on sections from one representative well/set, therefore, the final number of BrdU positive cells was multiplied by 6 (the number of representative wells) to estimate the full count in the whole DG of the hippocampus (Chamaa et al., 2016, 2018a). To determine whether the decrease was ipsilateral, contralateral or bilateral to both DG, the number of BrdU positive cells was counted in the left contralateral and right ipsilateral DG of all groups. For the total BrdU count of each rat, BrdU positive cells in the dentate gyri ipsilateral and contralateral to the injection site were summed to obtain the total number of BrdU cells per rat. Quantification of the TH immune reactive (TH-ir) cells was done manually on 40× and 5 representative SN sections were chosen from each rat. TH was represented as the % of the intact Non-injected SN.

BrdU positive cells co-labeled with DCX were counted manually on 40× oil.

Optical density of CD11b/c and GFAP immunostaining was measured in the ipsilateral DG side and expressed as a percentage of the contralateral side (constant area = 125,490.76 μm^2^). Five sections were taken as representative from each rat.

### Statistical Analysis

All data were presented as the mean ± standard error of the mean (SEM) of measurements made on each experimental group at each time interval. Statistical analysis and plotting of figures were performed using Prism 6 GraphPad package (GraphPad Software, Inc., CA, United States). Repeated measures ANOVA with sphericity assumed was used to check for significant differences between the time points in the Rota rod test. For all the remaining data analysis, unpaired 2 tailed student *t*-test was used for determining statistical significance and *p* < 0.05 was considered the limit of statistical significance.

## Results

### Effect of Intranigral Injection of ET on Hippocampal Stem/Progenitor Cell Proliferation and Survival

**FIGURE 2:**
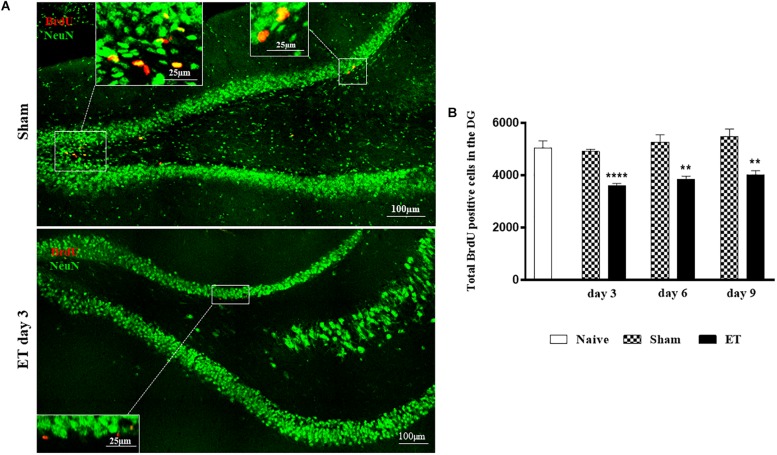
Intranigral injection of ET induced significant reduction of BrdU positive cells in the hippocampus. **(A)** Tile scans of the dentate gyrus of sham and ET-injected SN, at day 3, showing a decrease in proliferating cells in the ET injected rats. **(B)** Time course of the alteration of the count of total BrdU positive cells in both dentate gyri of each group showing a significant decrease in total BrdU positive cells at days 3, 6 and 9 post-injection (*n* = 5 each). Each bar represents the average of counted BrdU-positive cells ± SEM of both dentate gyri at the corresponding time interval. There was no statistical significance between shams (*n* = 5 each) and the naives (*n* = 5) at days 3, 6 and 9. The noted significance was made with reference to the shams (^*^) using student *t*-test; ^∗∗^*P* < 0.01; ^∗∗∗^*P* < 0.001.

The sum of total BrdU positive cells from both dentate gyri showed that the injection of ET in the SN lead to a prominent decrease in the total number of newly born proliferating hippocampal cells at day 3 post-injection (3609 cells ± 84) compared to sham (4911cells ± 89, *P* < 0.001) and naïve (5052 cells ± 272, *P* < 0.001) rats. This effect persisted until day 6 (3847 cells ± 120, *P* < 0.01) and day 9 (4032 cells ± 150, *P* < 0.01) compared to their respective shams (5279 cells ± 272 at day 6 and 5486 cells ± 287 at day 9) (Figures 2A,B). As expected, BrdU positive cells expressed double labeling with the proliferation marker ki67 on day 3 and not on day 6 (Supplementary Figure S1). Moreover, some of the BrdU positive cells started expressing the neuronal migration marker doublecortin (DCX) at days 6 and 9. Approximately 59.09 ± 1.69% of BrdU positive cells were co-labeled with DCX at day 9 (Supplementary Figure S2).

**FIGURE 3:**
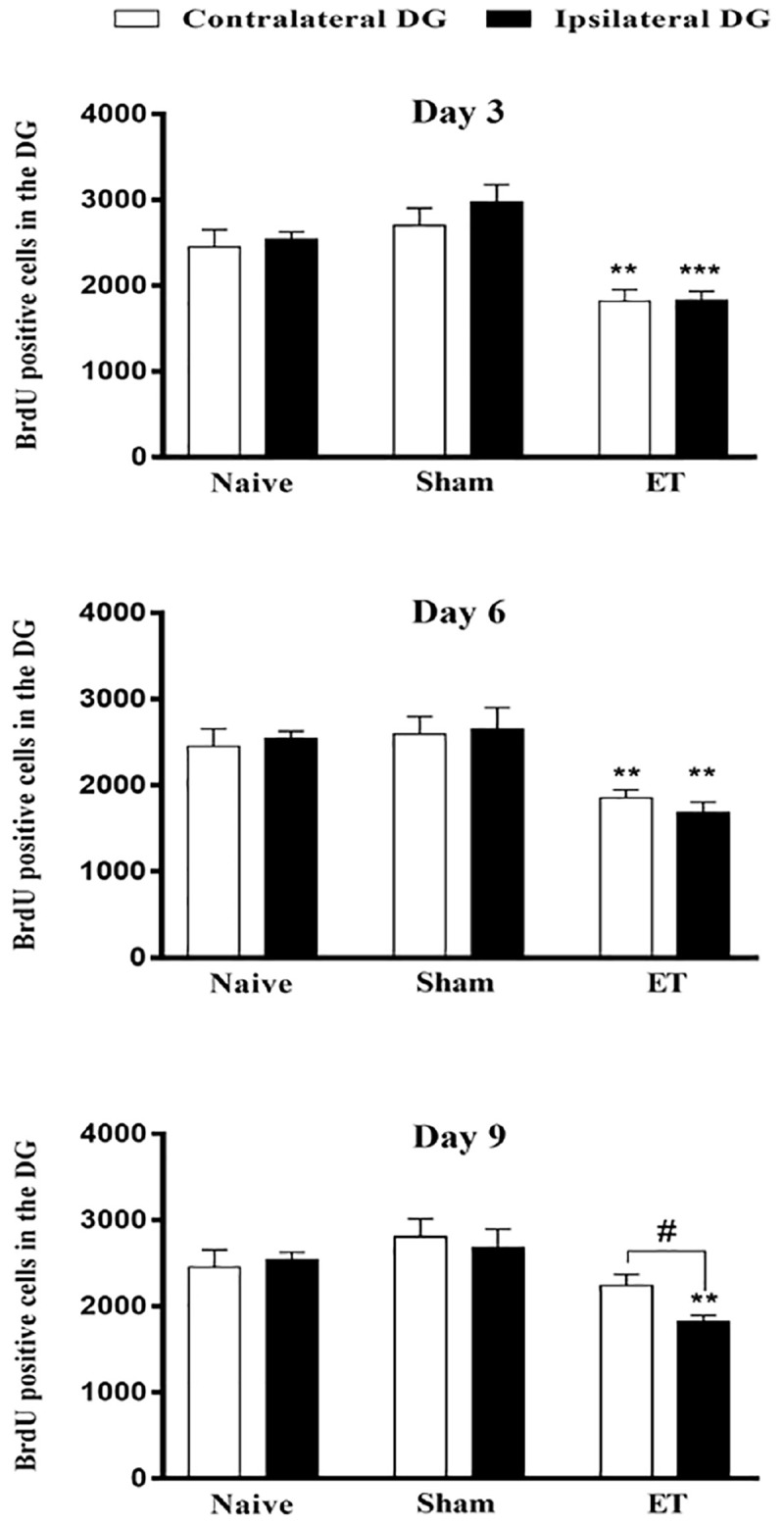
Bilateral attenuation of stem cell proliferation in the dentate gyri by intranigral injection of endotoxin. Quantification of BrdU positive cells in the ipsilateral and contralateral DG to the side of ET-intranigral injection, showing significant decreases in both sides. Each bar represents the average of counted BrdU-positive cells ± SEM of each dentate gyrus (contralateral or ipsilateral separately with reference to injected side). Measurements of significance between the ipsilateral and contralateral DG of ET is indicated by # and with their reference to sham by ^*^. There was no significant difference between sham and naïve at all-time points, using student *t*-test; ${}^{\#}$*P* < 0.01; ^∗∗^*P* < 0.01; ^∗∗∗^*P* < 0.001. Paired student *t*-test was used to determine significance between ipsilateral and contralateral counts within the same group.

The number of BrdU positive cells in the DG ipsilateral (1828.5 cells ± 105.8) and contralateral (1818 cells ± 135.45) to the injection site showed that the decrease in BrdU was bilateral at day 3 post-injection as compared to ipsilateral (2981.76 cells ± 197.27, *P* < 0.001) and contralateral DG (2699.52 cells ± 206.75, *P* < 0.01) of sham. This was also the case on day 6 where the differences in the number of BrdU positive cells in the ipsilateral (1682 cells ± 121.5) and contralateral (1851.6 cells ± 97.15) DG of ET were statistically significant compared to sham ipsilateral (2650 cells ± 251.05, *P* < 0.01) and contralateral DG (2594 cells ± 205.01, *P* < 0.01), respectively. As for day 9, there was a persistent significant (*P* = 0.05) decrease in BrdU positive cells in the ipsilateral DG (1821.6 cell ± 74.8) as compared to the contralateral DG (2236.8 cells ± 134.58) (Figure 3).

### Effect of Intranigral Injection of ET on Novel Arm Exploration in the Y-Maze

**FIGURE 4:**
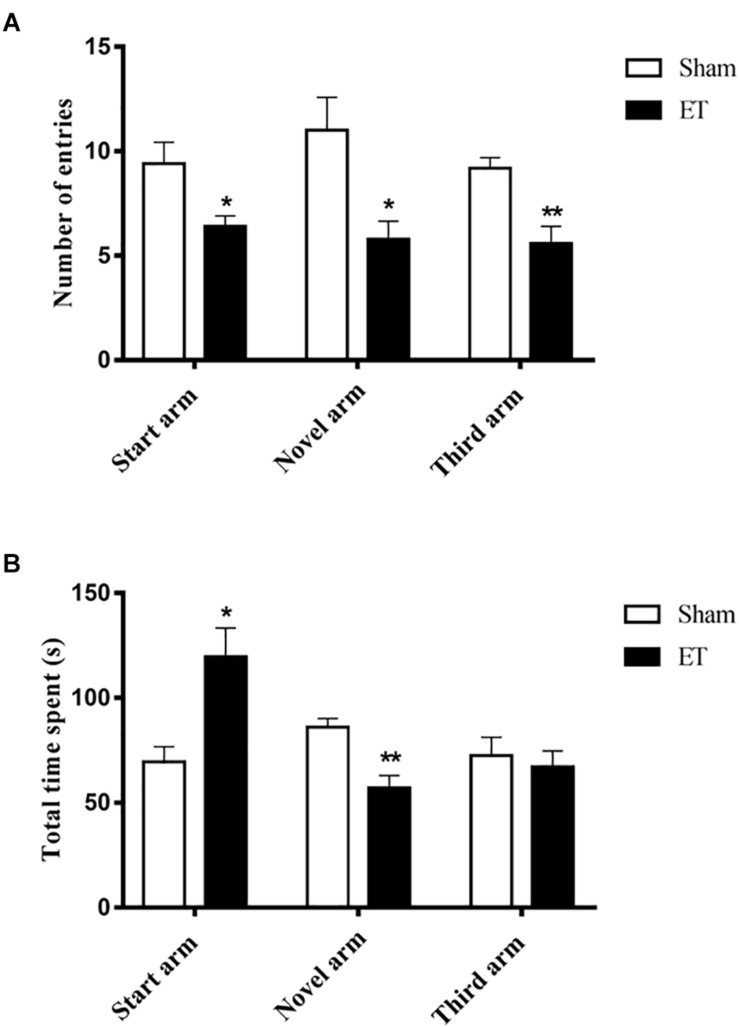
Decreased Novel arm exploration in the Y-maze post–intranigral inflammation. **(A)** A significant decrease in the number of entries to each of the three arms by the ET injected rats compared to sham. **(B)** A significant increase in the total time spent in the start arm versus a significant decrease in the novel arm recorded by ET injected rats. Statistical significance was performed using student *t*-test; ^∗∗^*P* < 0.01.

Results from the Y-maze showed that the number of entries to the start (6.4 entries ± 0.5), novel (5.8 entries ± 0.9) and third (5.6 entries ± 0.8) arms were significantly lower for ET-injected rats compared to sham (9.4 entries ± 1, *P* < 0.05), (11 entries ± 1.6, *P* < 0.05) and (9.2 entries ± 0.5, *P* < 0.01), respectively (Figure 4A). Moreover, the total time spent by ET- injected rats in the novel arm (57 s ± 5.99) was significantly lower than that of sham rats (86 s ± 4.2, *P* < 0.01). This was associated with a significant increase in the total time spent in the start arm by the ET-injected rats (119.67 s ± 13.66) in comparison to sham (69.4 s ± 7.2, *P* < 0.05) (Figure 4B).

### Effect of Intranigral Injection of ET on Hippocampal Glial Cells

**FIGURE 5:**
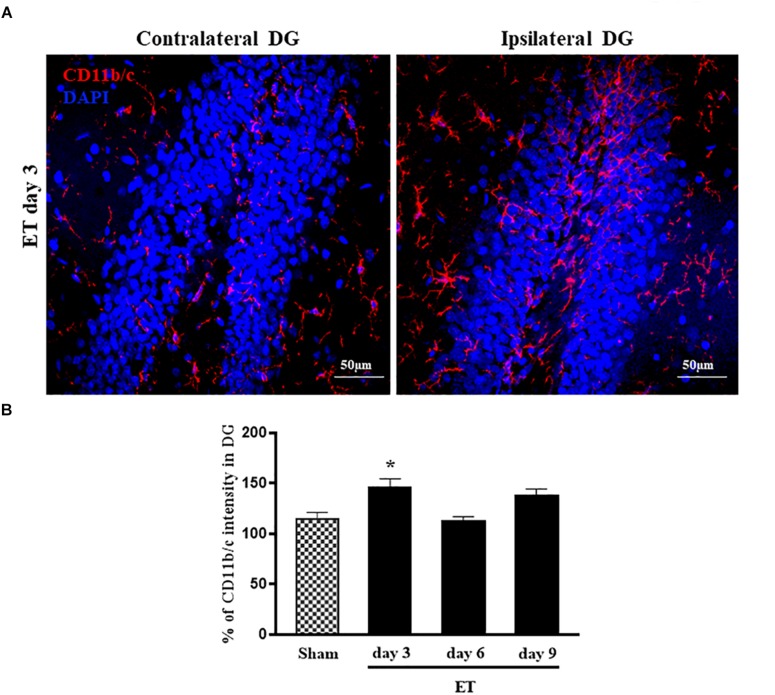
Microglial activation/proliferation in the dentate gyrus following ET injection. **(A)** Representative photomicrographs of hippocampal sections stained with CD11b/c and DAPI, showing more frequent microglial cells in the side ipsilateral to the ET-injected SN as compared to the contralateral DG. The confocal images were taken at 40× oil objective. **(B)** CD11b/c ipsilateral signal intensity (% of contralateral DG) of ET day3 (*n* = 5), day 6 (*n* = 5), day 9 (*n* = 5) and sham (*n* = 4). The significance of differences was determined using ANOVA followed by Bonferroni’s multiple comparison test; ^*^P < 0.05.

The signal intensity of the microglial marker, CD11b/c-staining was quantified in the dentate gyri of rats that were injected with either ET or saline in their SN and the obtained results were presented as % of the ipsilateral versus contralateral DG, with reference to the injected SM. Three days after ET injection, the ipsilateral DG had a significantly higher increase in % intensity of CD11b/c (145.6% ± 9.23) as compared to sham (107.98% ± 3.51, *P* < 0.05). This increase subsided by days 6 and 9 (Figures 5A,B). It should be noted that the microglia (MG) in the hippocampus, at all time-points, were ramified with no evidence of amoeboid activated configuration. It is worth noting, also, that no evident astrocytic activation was noticed in the DG throughout the observation period (Supplementary Figure S2).

### Effects of Intranigral Injection of Endotoxin on Dopaminergic Neurons

**FIGURE 6:**
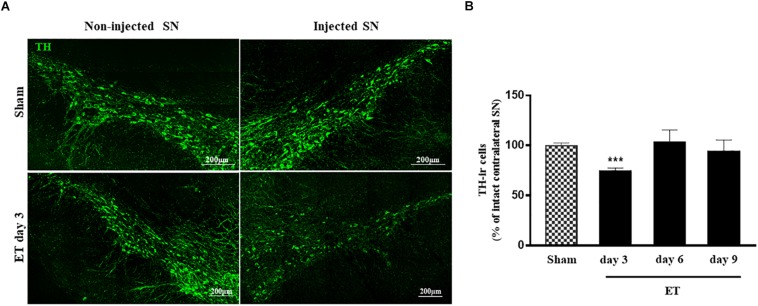
Transient reduction of TH positive neurons in the injected substantia nigra. **(A)** Tile scans of the injected (right) and non-injected (left) SN stained for TH in sham and ET injected rats showing a decrease in the number of TH-ir cells in the injected side of SN at day 3 only. **(B)** Bar charts representing the percent variations of TH positive cells count in sham (*n* = 5) and ET-injected rats at different time intervals (*n* = 5 at each time point). A significant decrease is noted at day 3 (*P* < 0.001) post-injection. Student *t*-test was used to determine statistical significance.

ET injection resulted in a significant decrease in the percentage of TH positive cells in the injected SN at day 3 (74.61% ± 2.97, *P* < 0.05) compared to sham (99.7% ± 2.9). This decrease was followed by recovery observed at days 6 (103% ± 12.59) and 9 (93.75% ± 11.72) (Figures 6A,B).

Moreover, microglial and astrocytic activation were evident at day 3 post–injection by the presence of amoeboid MG in the SN injected with ET as compared to the Non-injected side and to the saline injected SN of sham rats (data not shown).

### Effect of Intranigral Injection of Endotoxin on Motor Coordination

**FIGURE 7:**
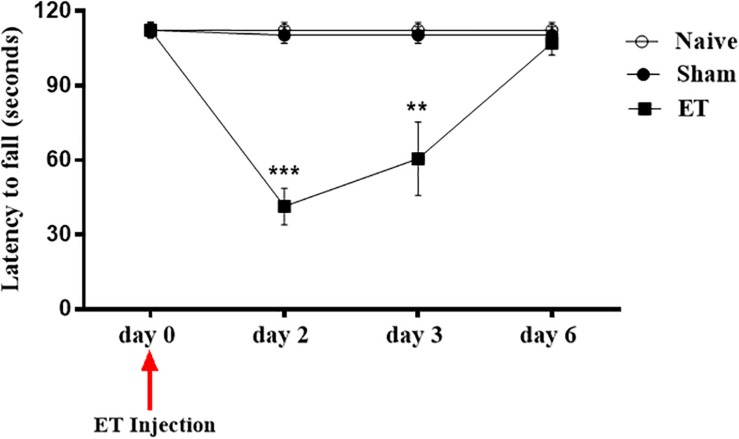
Intranigral ET injection induced significant alterations in rat’s motor behavior. Motor Coordination task performance. Two days following the intranigral ET injection, the rats’ (*n* = 12) latency to fall was significantly decreased compared to sham (*n* = 15) and naïve (*n* = 13). By day 6, ET injected rats (*n* = 7) had a complete recovery in performance. Repeated measure ANOVA with sphericity assumed was used to determine statistical significance between the time points [*F*(2, 4) = 7.144, *P* = 0.048]. ^∗∗^*P* < 0.01; ^∗∗∗^*P* < 0.001.

Motor coordination and balance were affected during the first three days after ET injection in the SN. The Rota rod test showed a significant decrease in the latency to fall off the rotating rod in the ET injected rats at days 2 (41.41 s ± 7.31; *P* < 0.0001) and 3 (60.61 s ± 14.76; *P* = 0.002) post- ET injection when compared to sham group (101.3 s ± 4.21). This was later followed by a trend to recovery at day 6 post–injection (Figure 7).

## Discussion

The results of the present study demonstrate that a single intranigral injection of endotoxin provoked a sustained and significant decrease in the proliferation, but not the survival, of NSPCs in the DG of the hippocampus over a period of 9 days. The ET injection doesn’t seem to affect survival as the BrdU positive cell counts of the ET injected rats at days 3, 6 and 9 were comparable to each other. In addition, the resulting inflammation from this injection caused localized microgliosis and a transient decrease in TH positive cells that was paralleled by contralateral motor deficits.

Previous studies of the effects of SN lesions on hippocampal NSPCs proliferation have been traditionally based on the use of toxins, such as MPTP and 6-OHDA that can produce a loss of dopaminergic neurons without mimicking the actual process of SN degeneration seen in PD (Suzuki et al., 2010; Salvi et al., 2016). Using inflammogens such as ET, provides a more plausible simulation of pathological situations, since inflammation (overt and/or discrete) is a commonly involved process in neurodegenerative diseases. Our findings present new evidence for altering stem/progenitor cell proliferation at the level of the hippocampus following acute and moderate inflammation in the SN, the region mainly affected in Parkinson disease. To our knowledge, this is the first report on the effect of reversible inflammation in the SN on NSPC proliferation in the DG.

The remote effects of the transient and localized nigral inflammation were translated by a decrease in newly born hippocampal cells over a period of 9 days. This is in line with a previous study which showed that similar intranigral injections of ET caused hippocampal oxidative stress-damage and subsequent cognitive impairment (Hritcu and Ciobica, 2013). The effect was restricted to proliferation and not survival as we would expect further decrease in BrdU positive cells at days 6 and 9 had survival been affected. Moreover, our data showed that the decrease in BrdU positive cells was distributed almost equally in both the ipsilateral and contralateral DG at days 3 and 6 after ET injection. The observed bilateral effect may reflect the complexity and redundant circuitry characteristics of the limbic connections. The hippocampus of the rat might differ in organization and laterality from other species, as it does not exhibit asymmetry in response to stimuli or events such as long-term potentiation (Martin et al., 2019). The observed bilateral decrease in neural stem cells could have been driven either by the inter-hippocampal connections and/or by the contralateral connections of the VTA, which projects 10% of the dopaminergic neurons to the contralateral hippocampal formation (Gasbarri et al., 1994; Hoglinger et al., 2014; McNamara et al., 2014). There are two distinct multi-synaptic VTA-hippocampal pathways in rodents that are similarly expressed in humans. Moreover, there is a direct circuitry from the SN to the hippocampus and vice-versa (Lisman and Grace, 2005). However, these connections are not completely delineated, and their functions are still being investigated. A unilateral injection in the mesolimbic system would not necessarily have ipsilateral effects on the brain. There has been evidence that there are more contralateral than ipsilateral changes in brains affected by Parkinson disease (Kempster et al., 1989; Sterling et al., 2013; Caligiuri et al., 2016). The basal ganglia, for example, are affected bilaterally regardless of the SN side of onset (Geng et al., 2006). Moreover, a unilateral lesion to the hippocampus was shown to affect the dopaminergic supply bilaterally (Uehara et al., 2000). Therefore, much is still needed to be known about the SN/VTA- hippocampal circuitry and laterality and how such connections affect each other intrinsically and under suppression or activation. It is worth noting that the injection site we opted for was near the VTA and the dopaminergic cells of the VTA were also compromised at day 3. Therefore, it is likely to expect a bilateral effect mediated by the VTA as well. Although this type of intranigral injection has been used by several authors to produce local and unilateral block of dopaminergic neurons, a possible spreading to the contralateral VTA might be envisaged to explain the bilateral effects observed at hippocampal level.

The decrease in hippocampal proliferating cells was associated with the transient decrease in TH positive cells seen at day 3 post ET injection in the SN. Given the important direct and indirect dopaminergic projections to the hippocampus and their potential trophic role, alterations in dopaminergic cells could affect the neurogenic niche at the hippocampus (Salvi et al., 2016). In this context, there has been growing evidence that dopaminergic enhancing compounds such as levodopa or DA agonists have the potential to boost neurogenesis in the SVZ and the hippocampus (Winner et al., 2009; McNamara et al., 2014). Dopamine is an important regulator of adult hippocampal neurogenesis and plays a role in memory circuitry of novel events mainly through the D1-and D-2 Like receptors (Freundlieb et al., 2006; Duszkiewicz et al., 2018). Using agonists to stimulate these receptors has proven to boost the depressed neurogenesis in PD or dopamine depletion models (Takamura et al., 2014; Salvi et al., 2016). Moreover, highly proliferative cells in the subependymal zone and the DG were shown to express dopamine receptors and receive dopaminergic afferents (Hoglinger et al., 2004). Furthermore, experimental depletion of dopamine in rodents decreases the proliferation of precursor cells in both the SVZ and the Sub granular zone of the DG (Hoglinger et al., 2004). Consequently, the number of proliferating neural precursor cells have been shown to be reduced in postmortem brains of PD patients (Regensburger et al., 2014). In addition, it has been reported before that the inflammatory damage caused by ET is restricted to dopaminergic neurons while maintaining the integrity of GABAergic and serotonergic neurons in the vicinity of the injection (Herrera et al., 2000). Therefore, the effect on stem cells and memory performance might have been mediated by the decrease of dopaminergic input to the hippocampus (Du et al., 2016; Salvi et al., 2016; Duszkiewicz et al., 2018).

The rotarod and Y-maze tests were used to assess motor coordination and exploratory behavior, respectively. The ET rats displayed less latency to fall of the rotating rod reflecting motor coordination imbalance. Moreover, ET injection in the SN induced a decrease in exploratory behavior of the rats as assessed by the Y-maze test. We noticed that the ET-injected rats had less tendency to explore the novel arm and this was detected by fewer entries with a decreased total time spent in that arm as well. In sham animals, the number of entries and the time spent showed a trend to increase for the new arm; while in rats treated with ET the decrease in number of entries can reflect a decrease in locomotor activity. However, the further shortening of time spent in the novel arm might reflect a decrease in exploratory behavior and novelty detection. It is known that dopaminergic system connections to the hippocampus play an important role in exploratory behavior, novel experiences and memory retention (Du et al., 2016; Duszkiewicz et al., 2018).

These results prompted us to investigate whether glial cells could be involved in this remote response. The signal intensity of the microglial cell marker, CD11b/c, was significantly increased in the DG ipsilateral to the ET-injected SN, but without exhibiting an amoeboid morphology. It is worth noting, here, that the role of microglia is not just restricted to immunological activation and surveillance, but under normal conditions, their role further extends to synaptic pruning, nurturing neural stem cells and regulation of neurogenesis (Siskova and Tremblay, 2013; Wake et al., 2013). Microglial activation has been documented in PET studies in PD patients even in their earlier stages of the disease and it shows a marked extra-striatal spreading in their brains (Terada et al., 2016). The transient disruption of the function of dopaminergic synapses in the hippocampus may be associated with the apparent moderate activation of microglia, which in turn may affect the decrease in NSPCs proliferation.

ET has been well characterized as an effective initiator of nigrostriatal degeneration and a potent activator of microglia, whether administered directly in the SN or intraperitoneally (Iravani et al., 2005; Qin et al., 2007; Choi et al., 2009; Hoban et al., 2013; Reinert et al., 2014; Valero et al., 2014). We showed that a low concentration of ET induced a transient but significant decrease in TH-ir neurons in the SN, which was restricted to the injected side. This comes in agreement with numerous reports that have used a similar concentration of ET and noted a decrease in TH-ir cells at comparable time points (Herrera et al., 2000; Castano et al., 2002; Santiago et al., 2010). However, unlike some studies, we did not observe an irreversible loss of the SN dopaminergic neurons. This discrepancy could be attributed to differences in experimental settings such as, gender and strains of rats and the reagents that were used in the different studies. It should be noted, also, that there are some inconstancies in the literature concerning the reported effects of such ET intranigral injection on SN neurons and behavioral testing (Herrera et al., 2000; Santiago et al., 2010). The fast recovery of TH that we saw by day 6 could indicate that the TH-ir neurons that were not seen at day 3 did not necessarily undergo cell death. Tyrosine hydroxylase is the rate-limiting enzyme that converts tyrosine to L-DOPA (L-3, 4 dihydroxyphenylalanine). Therefore, the decrease in TH could be explained by a transient dysregulation in its expression that may have been induced by the localized and moderate inflammation resulting from ET injection (Zhong et al., 2015). Moreover, the transient change in the number of TH-ir neurons may be attributed to the mild dosage of ET that was administered. Furthermore, we noted that the decrease in TH-ir cell counts was associated with microglial activation manifested by the presence of amoeboid microglia restricted to the injected SN. The amoeboid microglial structure is a typical indication of activation that was considered by previous studies as a determinant process involved in inflammation-driven neurodegeneration (Gao et al., 2002; Hoban et al., 2013; Reinert et al., 2014).

Our results demonstrate that a low dose of intranigral ET injection causes changes in the SN that leads to impaired NSPCs proliferation in the hippocampus. The described ET model appears to induce moderate and reversible effects on the SN neurons, unlike the irreversible and considerable effects seen with the use of the 6-OHDA (Suzuki et al., 2010) or MPTP (Salvi et al., 2016) models. These findings suggest that immune stimulation by intranigral LPS injection may offer a good *in vivo* model for studying the selective effects of moderate and transient inflammatory reactions on the dopaminergic systems. They further support that neural stem cell proliferation in the DG and exploratory behavior may be, in part, controlled by the dopaminergic midbrain input (Duszkiewicz et al., 2018).

In conclusion, this study demonstrates that moderate and localized neuroinflammation can have remote effects on other brain areas. Although inflammation in the SN may not be the primary cause of Parkinson disease or other neurodegenerative diseases, better understanding of its effects could provide new insights into the pathologic progression of neurodegenerative diseases and its association with cognitive impairment.

## Data Availability

All data are provided in full in the results section of this manuscript.

## Ethics Statement

This study was carried out in accordance with the recommendations and guidelines of the Institutional Animal Care and Use Committee. The protocol was approved by the Institutional Animal Care and Use Committee.

## Author Contributions

BD, FC, NS, and WA-K contributed to the project design and execution of experiments, analysis of results, and writing of manuscript. EA-C, NS, and WA-K critically revised and edited the manuscript. All authors have read and approved the final draft.

## Conflict of Interest Statement

The authors declare that the research was conducted in the absence of any commercial or financial relationships that could be construed as a potential conflict of interest.

## Supplementary Material

FIGURE S1 Co-labeling analysis for BrdU positive cells with Ki67 and DCX. **(A)** Representative photomicrographs of hippocampal sections showing co-labeling of BrdU-DCX at days 6 and 9, but not day 3. **(B)** Representative photomicrographs of hippocampal sections showing co-labeling of Ki67-BrdU positive cells at day 3. **(C)** Total BrdU positive cells ±SD and BrdU/DCX co-labeled ±SD cells in the DG at day 9.

FIGURE S2 Effect of intranigral ET injection on astrocytes in the DG. **(A)** GFAP in the DG of sham and ET. **(B)** Signal intensity of GFAP in the DG of sham and ET.

## Abbreviations

DG: dentate gyrus
ET: endotoxin
ICV: intracerebroventricular
IP: intraperitoneal
NSPCs: neural stem/progenitor cells
PD: Parkinson disease
SN: substantia nigra
TH: tyrosine hydroxylase.
