# Supplementary figures and images for: Intranigral Injection of Endotoxin Suppresses Proliferation of Hippocampal Progenitor Cells
Source: Front Neurosci. 2019 Jul 3;13:687. doi: 10.3389/fnins.2019.00687 (PMC6616074; doi:10.3389/fnins.2019.00687)

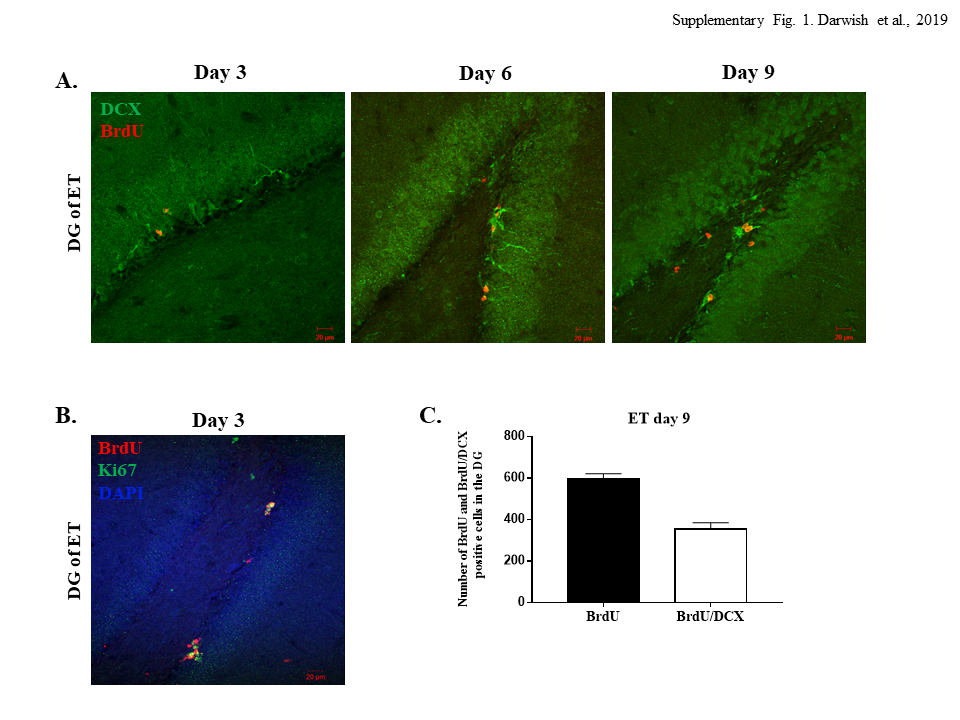

Supplement: FIGURE S1 — Co-labeling analysis for BrdU positive cells with Ki67 and DCX. (A) Representative photomicrographs of hippocampal sections showing co-labeling of BrdU-DCX at days 6 and 9, but not day 3. (B) Representative photomicrographs of hippocampal sections showing co-labeling of Ki67-BrdU positive cells at day 3. (C) Total BrdU positive cells ±SD and BrdU/DCX co-labeled ±SD cells in the DG at day 9. [file Image_1.TIF]

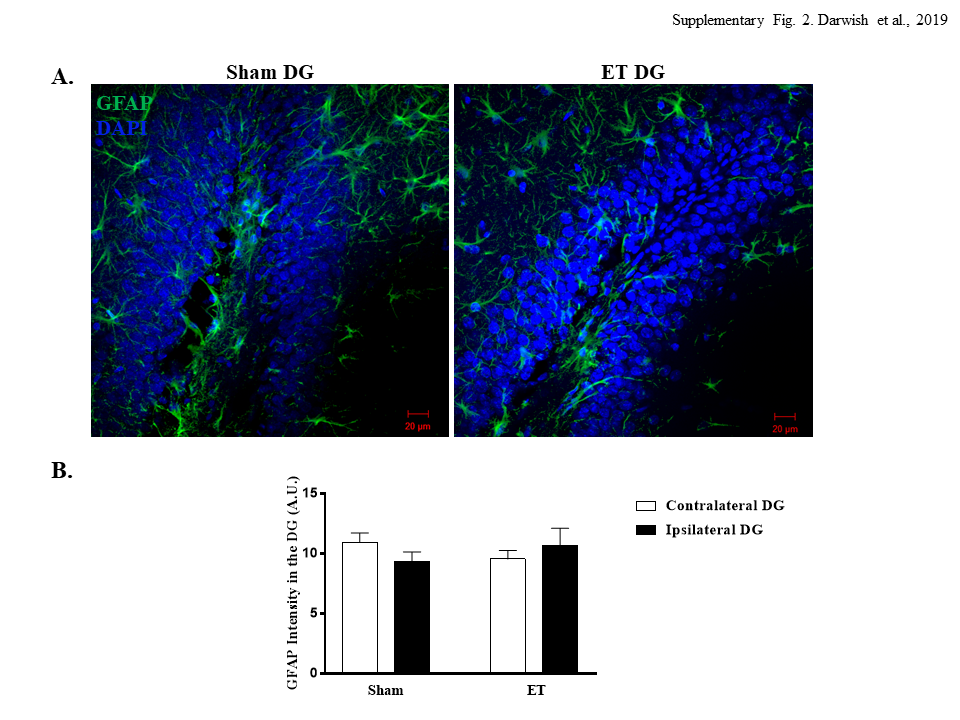

Supplement: FIGURE S2 — Effect of intranigral ET injection on astrocytes in the DG. (A) GFAP in the DG of sham and ET. (B) Signal intensity of GFAP in the DG of sham and ET. [file Image_2.TIF]
